# Impact of late effects after treatment for bladder cancer with radical cystectomy on Quality of life: a case-control study

**DOI:** 10.2340/1651-226X.2025.41040

**Published:** 2025-01-08

**Authors:** Rikke V. Milling, Ninna K. Nielsen, Charlotte Graugaard-Jensen, Peter Christensen, Helle Pappot, Jørgen B. Jensen

**Affiliations:** a Department of Urology, Aarhus University Hospital, Aarhus, Denmark; b Department of Clinical Medicine, Aarhus University, Aarhus, Denmark; c Department of Surgery,Aarhus University Hospital, Aarhus, Denmark; d Department of Oncology, Rigshospitalet, Copenhagen, Denmark

**Keywords:** Bladder cancer, late effects, quality of life, radical cystectomy

## Abstract

**Background and purpose:**

The gold standard when treating muscle-invasive bladder cancer (MIBC) is radical cystectomy (RC), a procedure that holds the potential to affect the function of several pelvic organs, causing an impact on the patient’s Quality of Life (QoL). Knowledge of the late effects following bladder cancer and treatment with RC is sparse. The aim is to describe the incidence of late effects and to investigate the impact on QoL.

**Methods:**

A cross-sectional study using register data in combination with a questionnaire, measuring pelvic organ specific symptoms to treatment and QoL. MIBC patients diagnosed between 2015 and 2020 and able to receive digital mail was invited. For each MIBC patient, 6 age- and gender matched controls were invited. QoL was measured using EORTC-QLQ-C30. The MIBC specific EORTC-QLQ-BLM30 was administered to cases only.

**Results:**

A total of 628 (54.3%) MIBC patients and their 1,204 (37.3%) matched controls responded. Median age was 73. Mean time since RC was 4.9 (SD 2.1) years. Scoring of the functional items on EORTC-QLQ-C30 and overall QoL were similar for cases and controls. Regarding late effects, similar responses were seen on questionnaire data when comparing cases and controls. On registry data, a higher risk of infections and hydronephrosis were seen for cases. A strong correlation between fatigue and impaired QoL was identified.

**Interpretation:**

MIBC patients were more often diagnosed with late effects such as infections and hydronephrosis, compared to controls. In spite of this, MIBC patients overall QoL was equal to that of the controls.

## Introduction

Bladder cancer is the 10th most common cancer, affecting males and females in a 4:1 ratio. Bladder cancer can be either non-muscle invasive (NMIBC) or muscle invasive (MIBC). The gold standard when treating high risk NMIBC and MIBC is radical cystectomy (RC) with urinary diversion [1].

In addition to the bladder being removed and a conduit being created from the bowel, RC includes removal of the prostate and the seminal vesicles for men, and for most women, the removal of the anterior vaginal wall, the uterus, and ovaries [2, 3]. Thus, the surgery has the potential to affect or disrupt the function of several pelvic organs, causing a severe impact on the patient’s life, which to some extend can be measured by surveying quality of life (QoL) [4].

Various questionnaires have been developed to measure QoL. In bladder cancer research, the most frequently used generic questionnaires are the European Organisation for Research and Treatment of Cancer’s (EORTC) EORTC-QLQ-C30 [5] and the Functional Assessment of Cancer Therapy’s (FACT) FACT-G [6]. These generic questionnaires can be combined with bladder cancer specific questionnaires, most frequently used is the EORTC-QLQ-BLM30 [7] and the FACT-Bl [8]. Furthermore, symptom-specific questionnaires measuring for example sexual health has been developed and the EORTC-SHQ-C22 is used to measure sexual health among cancer patients [9].

Using these instruments, several studies have demonstrated the impact of bladder cancer and its treatment on QoL, which often leads to more deterioration in QoL compared to other pelvic organ cancers [10–12].

One explanation for this, could be the long-term consequences of treatment, also known as late effects. Late effects are defined as complications occurring during treatment and persisting longer than 3 months after the intervention [13]. As the amount of long-term survivors increase, attention towards both research and treatment of late effects has grown. Although the mortality associated with bladder cancer has hardly improved much during the past decade, long-term survivors exist, and they report that the treatment has impacted their lives [14]. Still, knowledge about late effects among bladder cancer survivors is sparse.

Therefore, the aim of this study is to describe the incidence of late effects following treatment for bladder cancer with RC and to investigate the impact on the patients’ QoL.

## Methods

### Patient selection

This study is a national cross-sectional register study in combination with a questionnaire survey. The Danish National Patient Registry (DNPR) was used to identify the study population [15]. We included all Bladder Cancer patients diagnosed between 1st of January 2015 and 31st of December 2020 treated with RC and urinary diversion and still alive on the 17th of August 2022.

For each bladder cancer patient (cases), 6 age- and gender-matched controls were identified in the DNPR.

Demographic data on age, comorbidities, peri- and post-operative complications were obtained from the Central Person Registry (CPR) and DNPR. Additionally, the DNPR was used to obtain details on treatment using the International Classification of Disease (ICD) 10 codes (ICD10-code). The tumour-node-metastasis (TNM) stages were obtained from the Danish Cancer Registry (CAR). The diagnosis of Alzheimer’s or dementia registered in the DNPR served as a condition-specific exclusion criteria, allowing for the presence of other malignancies. SurveyXact was used to distribute the electronic questionnaire. The questionnaire was only distributed to those receiving digital mail from the authorities through the Danish digital mailbox system (E-boks). Mails distributed to the digital mailbox are not sent to an email-address, but to the social security number of the citizen. The invitations were distributed to the entire cohort at once. If no response had been registered after 9 days, a reminder was sent.

The study adheres to the General Data Protection Regulations and was reported to the Danish Data Protection Agency (Central Denmark Region, record number: 1-16-02-394-21). In accordance with the Danish legislation, no separate ethical approval was required.

### Questionnaires

The survey consisted of the following questionnaires:

The generic QoL questionnaire EORTC-QLQ-C30 and the sexual health questionnaire EORTC-SHQ-C22 were administered to all participants, and the bladder cancer specific questionnaire EORTC-QLQ-BLM30 to cases only. All EORTC questionnaires are scored on a scale from 0 to 100. On EORTC-QLQ-C30, a high score on functional items represents a high QoL. On all EORTC questionnaires, a high score on symptom items represents a high level of symptomatology [5, 9]. To elaborate on erectile function, the International Index of Erectile Function (IIEF5) was included [16]. Questions 2–6 were only administered to sexually active responders.

Furthermore, the Bristol Stool Form Scale was included to add a measurements of bowel function on other questionnaires [17].

In addition, selected questions from the National Cancer Institute’s (NCI) PRO Common Terminology Criteria for Adverse Events (CTCAE) library covering anxiety, depression, concentration, and memory loss were included. These questions were scored from 0 to 4, and a high score indicated high symptomatology.

After each section of questions (urological, sexual, gastrointestinal), the patients were asked to evaluate the impact of their experienced problems on their QoL on a 4-point scale; ‘Not at all’, ‘A little’, ‘Some’, ‘A lot’, this served as an anchor question.

Prior to distribution of the questionnaire, a pilot-study was conducted inviting eight patients who attended the standard post-cystectomy follow-up programme. They completed the questionnaires and were afterwards interviewed about the content and questions asked using a semi-structured approach (For interview-guide, see Supplementary Table 1).

### DNPR data

For cases, late effects were defined as diagnoses dated to occur after the date of cystectomy. The index date of a case was applied to their matched controls, thus including only diagnoses registered after the index date.

### Statistical analysis

Statistical analyses were conducted using *R* version 4.2.2. [18].

The EORTC questionnaires were scored and analysed according to guidelines, omitting missing data [5, 9, 19]. Missing data on the additional questionnaires are presented when relevant and omitted when contributing to a multi-item analysis, removing the multi-item score.

In the analysis of descriptive statistics, the mean and standard deviation were applied to data following a normal distribution, while the median and quartiles (Q1, Q3) were utilised for non-normally distributed data. Frequencies and percentages are presented, especially when reporting on Patient Reported Outcome Measures (PROM) results. Wilcoxon signed-rank test and Spearman’s correlations were applied when testing for correlations between PROM symptoms and the Global Health Scale on EORTC-QLQ-C30.

## Results

**Figure 1:**
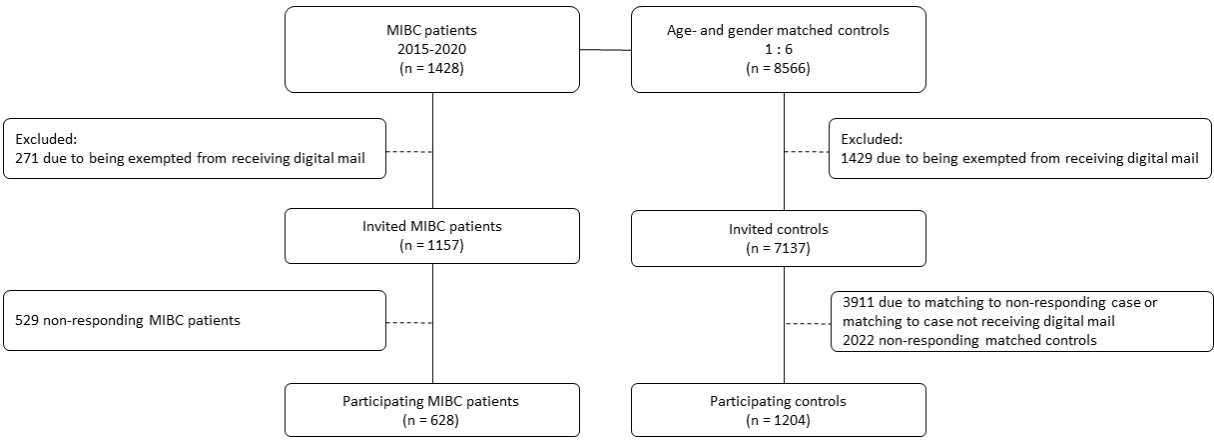
Flowchart of study inclusion process.

A total of 1,428 bladder cancer patients treated with RC were identified. A total of 271 (19%) patients were excluded due to being exempted from receiving digital mail. A total of 8,566 controls were identified, as two cases had only five matched controls. Among controls, 1,429 (17%) were excluded due to being exempted from receiving digital mail (Figure 1).

Of the invited 1,157 cases, 628 (54.3%) responded. Of their 3,226 invited controls, 1,204 (37.3%) responded. The overall response rate was 41.8%.

Matching for age and sex, we found similar age- and sex proportions. Furthermore, the cohorts matched according to BMI and comorbidity burden, measured by age adjusted Charlson comorbidity index. Cases were more likely to have a history of smoking, compared to controls (*p* < 0.001). Median age of responding cases was 73 (Q1: 67, Q3: 79). For non-responding cases, median age was 75 (Q1: 67, Q3: 81). Median age-adjusted Charlson Comorbidity Index (AA-CCI) was 4 (Q1: 3, Q3: 4) for responding cases and 4 (Q1: 3, Q3: 5) for non-responding cases.

Additional demographic characteristics of the cohort are listed in Table 1. Organ-confined BC was defined as carcinoma in situ (CIS) or T1–T2, N0, M0 at the time of cystectomy. Regarding patients with other pelvic organ cancers, we found 107 (17%) among cases and 96 (8%) among controls (*p* = 0.36). The most common pelvic cancers found were prostate cancer, found in 97 (15%) of cases and 79 (7%) of controls and rectal cancer, found in 7 (1%) of cases and 7 (0.5%) of controls.

**Table 1:** Study population characteristics.

Characteristic	Case	Control	*p*
	*N* = 628	*N* = 1204	
Age	73 (67, 79)	73 (67, 79)	0.93
Sex			0.99
Male	476 (76%)	913 (76%)	
Female	152 (24%)	291 (24%)	
BMI			0.75
<25	189 (30%)	362 (31%)	
25–30	323 (52%)	589 (50%)	
>30	112 (18%)	225 (19%)	
Smoking status			<0.001
Never	123 (20%)	481 (40%)	
Former	401 (64%)	589 (50%)	
Current	103 (16%)	118 (10%)	
Age adjusted CCI	4 (3, 4)	4 (3, 4)	0.42
Organ-confined BC	509 (81%)	-	
Time since surgery (Years)	4.9 (2.1)	-	
Cystectomy			
Neoadjuvant chemotherapy	283 (45%)	-	
Robot-assisted radical cystectomy	456 (73%)	-	

Mean (SD), Median (Q1, Q3), *n* (%)

CCI: Charlson comorbidity index; BC: Bladder cancer; BMI: Body mass index.

Scoring of the functional items on EORTC-QLQ-C30 and overall QoL were similar for cases and controls. The scores of the EORTC-QLQ-C30 are listed in Table 2. The scores on the functional items were high, indicating good QoL. Missing items to the EORTC-QLQ-C30 ranged from 0 to 3%.

**Table 2:** EORTC-QLQ-C30.

Characteristic	Case	Control
	*N* = 628	*N* = 1204
Global Health Scale	83.3 (66.7, 83.3)	83.3 (66.7, 91.7)
Physical Functioning	86.7 (73.3, 100)	93.3 (80, 100)
Role Functioning	100 (66.7, 100)	100 (83.3, 100)
Emotional Functioning	91.7 (83.3, 100)	91.7 (83.3, 100)
Cognitive Functioning	83.3 (83.3, 100)	100 (83.3, 100)
Social Functioning	83.3 (66.7, 100)	100 (83.3, 100)

Median (Q1, Q3)

The prevalence of late effects was higher and the subjective burden of urological problems on QoL was evaluated to be higher for cases compared to controls (*p* < 0.001). Scoring of the multi-item urostomy problems were a median of 11.1 (Q1: 5.55, Q3: 22.22), indicating mild symptomatology (Table 3).

**Table 3:** Urological late effects.

Characteristic	Case	Control
	*N* = 628	*N* = 1204
EORTC-QLQ-BLM30		
Urostomy problems	11.11 (5.55, 22.22)	-
Data from DNPR		
UTI	229 (36%)	150 (12%)
Urosepsis	153 (24%)	38 (3%)
Pyelonephritis	43 (7%)	13 (1%)
Hydronephrosis	64 (10%)	7 (0.5%)
LUTS	-	218 (18%)
Impact of urological problems on QoL		
Not at all	272 (43%)	679 (56%)
A little	242 (39%)	354 (29%)
Some	72 (11%)	68 (6%)
A lot	17 (3%)	15 (1%)
Missing	25 (4%)	88 (8%)

Median (Q1, Q3), *n* (%), UTI: Urinary Tract Infection.

DNPR: Danish National Patient Registry

The control cohort was more likely to be sexually active and the reasons for sexual inactivity differed significantly between the groups (*p* < 0.001), with erectile dysfunction being the most common reason for sexual inactivity among cases (Table 4). No differences in responses of the EORTC-QLQ-SH22 was found on either single- or multi-items. Scoring of the questionnaire were generally low, indicating mild symptomatology for both cases and controls. A difference was observed on the IIEF5, identifying a higher incidence of moderate and severe erectile dysfunction among sexually active cases (14%) compared to controls (6%). Additionally, the impact of sexual problems on QoL was significantly worse for cases compared to controls (*p* < 0.001).

**Table 4:** Sexual late effects.

Characteristic	Case	Control
	*N* = 628	*N* = 1204
Sexually active	139 (23%)	568 (51%)
Reasons for sexual inactivity		
Lack of desire	52 (11%)	76 (12%)
Erectile dysfunction	267 (54%)	174 (27%)
Loss of partner	9 (2%)	12 (2%)
Pain or discomfort	52 (11%)	153 (24%)
Other	83 (17%)	128 (20%)
Missing	26 (5%)	93 (15%)
EORTC-SHQ-C22		
Sexual activity	33.3 (33.3, 66.7)	33.3 (33.3, 66.7)
Decreased libido	33.3 (0, 33.3)	0 (0, 33.3)
Worries of incontinence during sexual activity	0 (0, 0)	0 (0, 0)
Impact of fatigue on sexual activity	33.3 (0, 33.3)	33.3 (0, 33.3)
Impact of treatment on sexual activity	66.7 (33.3, 100)	-
Partnership	33.3 (0, 66.7)	33.3 (0, 33.3)
Vaginal dryness	33.3 (0, 66.7)	33.3 (0, 66.7)
Confidence erection	100 (33.3, 100)	33.3 (33.3, 66.7)
Body image (male)	33.3 (0, 66.7)	-
Body image (female)	33.3 (0, 66.7)	-
Sexual satisfaction*	41.7 (20.8, 57.3)	25 (12.5, 37.5)
Sexual pain*	0 (0, 11.1)	0 (0, 0)
IIEF5 scoring		
Severe ED	48 (10%)	21 (3%)
Moderate ED	18 (4%)	27 (3%)
Mild to moderate ED	12 (3%)	57 (6%)
Mild ED	13 (3%)	134 (14.6%)
No ED	11 (2%)	202 (22%)
Missing	374 (78%)	472 (51%)
Data from DNPR		
Erectile dysfunction	55 (9%)	21 (2%)
Vaginal dryness	<5 (<1%)	<5 (<1%)
Decreased libido	-	-
Sexual counselling	-	-
Impact of sexual problems on QoL		
Not at all	206 (33%)	559 (46%)
A little	187 (30%)	373 (31%)
Some	148 (24%)	138 (11%)
A lot	53 (8%)	32 (3%)
Missing	34 (5%)	102 (9%)

ED: Erectile dysfunction.

* Multi-item, *n* (%), median (Q1, Q3)

DNPR: Danish National Patient Registry; IIEF: International Index of Erectile Function.

The symptoms measured on EORTC-QLQ-C30 showed that gastrointestinal problems were mild in both groups (Table 5). Still, the impact of gastrointestinal problems on QoL was significantly worse for cases compared to controls (*p* < 0.001).

**Table 5:** Gastrointestinal late effects.

Characteristic	Case	Control
	*N* = 628	*N* = 1204
EORTC-QLQ-C30		
Nausea and vomiting	0 (0, 0)	0 (0, 0)
Appetite loss	0 (0, 0)	0 (0,0)
Constipation	0 (0, 33.3)	0 (0, 33.3)
Diarrhoea	0 (0, 33.3)	0 (0, 33.3)
EORTC-QLQ-BLM30		
Abdominal bloating and flatulence	33.3 (16.7, 33.3)	-
Bristol stool chart score	4 (3, 4)	4 (3, 4)
Data from DNPR		
Nausea and vomiting	22 (4%)	20 (2%)
Appetite loss	<5 (<1%)	<5 (<1%)
Constipation	46 (7%)	58 (5%)
Diarrhoea	14 (2%)	23 (2%)
Parastomal hernia	22 (4%)	8 (<1%)
Incisional hernia	29 (5%)	12 (1%)
Impact of gastrointestinal problems on QoL		
Not at all	361 (57%)	834 (69%)
A little	167 (27%)	206 (17%)
Some	50 (8%)	44 (4%)
A lot	13 (2%)	14 (1%)
Missing	37 (6%)	106 (9%)

*Multi-item, Median (Q1, Q3), *n* (%)

DNPR: Danish National Patient Registry.

The scores on PRO-CTCAE were low in both groups, correlating with the low prevalence of diagnoses of anxiety and depression identified in DNPR (Table 6). Fatigue and insomnia measured on EORTC-QLQ-C30 were mild and similar in the two groups.

**Table 6:** Psychological late effects.

Characteristic	Case	Control
	*N* = 628	*N* = 1204
EORTC-QLQ-C30		
Fatigue	22.2 (11.1, 33.3)	22.2 (11.1, 33.3)
Insomnia	0 (0, 33.3)	0 (0, 33.3)
PRO-CTCAE		
Discouraged: Frequency	1.0 (1.0, 2.0)	1.0 (1.0, 2.0)
Discouraged: Severity	1.0 (1.0, 2.0)	1.0 (1.0, 1.0)
Discouraged: Impact	1.0 (1.0, 2.0)	1.0 (1.0, 1.0)
Sad: Frequency	1.0 (1.0, 2.0)	1.0 (1.0, 2.0)
Sad: Severity	1.0 (1.0, 2.0)	1.0 (1.0, 2.0)
Sad: Impact	1.0 (1.0, 2.0)	1.0 (1.0, 1.0)
Concentration: Frequency	1.0 (1.0, 2.0)	1.0 (1.0, 1.0)
Concentration: Impact	1.0 (1.0, 2.0)	1.0 (1.0, 1.0)
Memory: Severity	1.0 (1.0, 2.0)	1.0 (1.0, 2.0)
Memory: Impact	1.0 (1.0, 2.0)	1.0 (1.0, 1.0)
Anxious: Frequency	1.0 (1.0, 1.0)	1.0 (1.0, 1.0)
Anxious: Severity	1.0 (1.0, 1.0)	1.0 (1.0, 1.0)
Anxious: Impact	1.0 (1.0, 1.0)	1.0 (1.0, 1.0)
Data from DNPR		
Anxiety	<5 (<1%)	9 (<1%)
Depression	<5(<1%)	24 (2%)

*Median (Q1, Q3), *n* (%)

DNPR: Danish National Patient Registry; CTCAE: Common Terminology Criteria for Adverse Events.

Testing for correlations, we identified one strong correlation between fatigue and Global Health scale (r(149) = -0.62, *p* < 0.001). The other correlations tested were either very weak, weak, or moderate (See supplementary Table 2).

## Discussion

This study demonstrates that the overall QoL were equally high for cases with bladder cancer and controls. However, cases often found that their urinary, sexual, and bowel symptoms all had a higher impact on QoL than controls.

On EORTC-QLQ-C30, there was a tendency towards better physical functioning, cognitive functioning, and social functioning for controls compared to cases. However, the difference is estimated to be small for physical functioning and medium for social- and cognitive functioning [20], and is thus not interpreted to be of significance. The scoring on the questionnaire for cases are similar to that of other studies [10, 12] or even slightly higher [21]. Furthermore, the scores for both cases and controls are similar and even better than the reference data for all cancer patients. The Global Health Scale scores for both cases and controls are higher compared to the reference values for the general population [22]. This finding could potentially be explained by the gap theory or the happy survivor effect, described in psychological research [23, 24]. Both theories describe how accumulated experiences, surviving critical illness and older age can be linked to increased life satisfaction and improved QoL.

The formation of a urostomy, alters the urinary system for cases, resulting in large differences with regard to urological problems between cases and controls. Thus, interpreting the impact of the urostomy correctly by comparison is difficult. The scoring of urostomy problems in this study are similar or lower compared to that of other studies [25], indicating lesser symptomatology. As the urostomy complicates comparison, the search for late effects in DNPR focused on ICD10-codes that could occur in both cases and controls. We found that cases were more prone to develop infectious disease in their urinary tracts as well as hydronephrosis. Infections are a well-known risk following surgical procedures and strictures resulting in hydronephrosis are highly prevalent following RC [26]. The fact that the cases evaluated their urological symptoms to have a greater impact on their QoL, could therefore partially be explained by confirmation bias, where patients correlate their urological symptoms with negativity, as they have experienced great impact on their lives as a result of disease in their urinary tract.

In this study, 24% of cases were sexually active. Similarly, Jubber et al. found that 26% of patients treated with RC or radiotherapy were sexually active compared to 36% for patients treated with transurethral resection of bladder tumour (TURBT) and/or installation therapy [27]. It is well known that RC heavily influences erectile function and orgasmic ability, irrespective of attempting to conduct nerve-sparring surgery, which could explain the tendency to lesser sexual activity among cases compared to controls [28]. This hypothesis is supported by the results, with erectile dysfunction being the most common reason for sexual inactivity, particularly among cases compared to controls. Treatment is available for both men and women, but our data from DNPR shows that neither many responders have a ICD10-code of erectile dysfunction or vaginal dryness nor do they have a ICD10-code for sexual counselling. Considering how many patients report such problems on the questionnaire, our finding could indicate, that sexual problems are rarely discussed with the surgeon. Considering that cases evaluate the impact of sexual problems on QoL significantly worse than controls, our study supports the findings of other studies; the urologist should inform and initiate the conversation concerning sexual problems with the patients in order to reduce this burden [29, 30].

In this study, the PRO data suggests that gastrointestinal problems 5 years after cystectomy are generally mild and no different from that of controls. Interestingly, bladder cancer patients report a higher impact on QoL than controls. Other studies using different questionnaires have identified that flatulence and diarrhea are problems of concern for bladder cancer patients following RC [31, 32]. However, they also found that bowel function improves after surgery and becomes less significant to patients in the long term [33, 34]. The EORTC-QLQ-BLM30 multi-item on abdominal bloating and flatulence, diarrhoea, and constipation were similar to that of other studies [12, 14]. Thus, either the instrument applied fails to accurately describe the symptomatic burden of the patients, or the symptoms improve over time, but the memory of the problems remains with the patients, resulting in confirmation bias when the patient are asked to evaluate the impact of their bowel function on QoL.

The psychological late effects revealed that symptoms were generally mild and there were no differences in PROM data and DNPR data between cases and controls. These findings are surprising, as other studies have identified a correlation between mental health problems and impairment of QoL [35] and that the impact of bladder cancer on mental health often results in diagnoses of anxiety and depression, resulting in higher suicide rates [36]. The requirement for participants in our cohort to be alive and willing to answer the questionnaire may have influenced the psychological outcomes of this study. One could hypothesise, that those struggling with mental health problems might not be willing to participate, resulting in responder bias, which could explain our results. Another potential explanation could be that the median time since cystectomy in our study is 4.9 years. Optimal timing regarding evaluation of impact of symptoms on QoL is up for debate, but Kulaksizoglu and colleagues suggest waiting until after 12 months, based on their finding that patients often return to baseline within the first year [37]. Another study by Cerruto and colleagues found that patients typically improved to levels above baseline 7 years post-surgery [38]. Therefore, our findings might be influenced by the inclusion of long-term survivors who have endured the initial challenging years of the disease and are now less affected.

The differences in proportion of other pelvic organ cancers can mainly be contributed to the differences in prostate cancer, which potentially can be explained due to incidental findings during cystoprostatectomy. Thus, no true differences in the cohorts were identified, aside from tobacco history and the bladder cancer diagnosis, suggesting a well-matched study population.

This study has some limitations. Firstly, the cross-sectional design limits the possibility to conclude on the timely relation between symptoms and QoL. Secondly, selection bias could occur, as we chose to invite patients who had been diagnosed 3 years or more prior to being invited to participate. Thus, those heavily affected by the diagnosis and treatment within the first 3 years of diagnosis, have not been heard in this study. We speculate that the potential for bias in this study will affect our hypotheses towards the null. Additionally, non-responding cases and controls were generally older and slightly more comorbid than participants, which could potentially influence the results related to QoL. Furthermore, the difference in response rates between cases and controls might also introduce bias due to differential participation, limiting generalisability [39]. The strengths of this study include the size of the population and the ability to provide both subjective data in the form of PROMs and objective data from the DNPR, painting a more detailed picture of the burden experienced by the patients.

## Conclusion

This study found a higher risk of late effects such as infections and hydronephrosis for bladder cancer patients compared to a control population. Regarding sexual function, bladder cancer patients are more often experiencing erectile dysfunction and evaluate sexual problems to have higher impact on their QoL compared to controls. Although no higher scores of gastrointestinal problems were identified for cases, patients reported to be more affected by gastrointestinal problems compared to controls. Regarding psychological late effects, mild symptomatology was found, but especially fatigue seems to have a significant negative impact on QoL. Despite having a higher degree of symptomatology, bladder cancer patients’ overall QoL was equal to that of the controls.

## Supplementary Material



## Data Availability

The corresponding author is willing to provide the scripts used for data analysis upon request. Additionally, the corresponding author will fully cooperate with the editor or their assignees in explaining the data underlying the manuscript. However, due to Danish laws and regulations, the raw data from the Danish National Health Registries cannot be made available. Data from the questionnaires may be accessible in anonymised form, subject to approval by both the editors and the corresponding author, if the intended purpose is deemed reasonable and necessary.
