# Supplementary material for: Impact of late effects after treatment for bladder cancer with radical cystectomy on Quality of life: a case-control study
Source: Acta Oncol. 2025 Jan 8;64:41040. doi: 10.2340/1651-226X.2025.41040 (PMC11734305; doi:10.2340/1651-226X.2025.41040)
Supplement: Supplementary file 1 [file AO-64-41040-s1.pdf]

Supplementary material has been published as submitted. It has not been copyedited, or typeset by Acta Oncologica

Supplementary table 1:

**General questions for the semi-structured interview:**
1) Did you find any question difficult to understand?
2) Did you find any question difficult to answer?
3) Were any questions transgressive/taboo-breaking for you?
4) Were there any questions you missed and that you think could be relevant?
5) Is the layout good. Is the structure of the questions logical? Is the font size easily readable?
6) Do you have any additional comments about the questionnaire?
**For each of the treatment-specific questions, the patient was asked:**
a) Is the question easy/difficult to understand?
b) How did you find having to place yourself in one of the answering categories?
c) Do you have any suggestions on how any of the questions could be phrased better?

SUPPLEMENTARY TABLE 2.

y	x	rho	p	Spearman's
C30$QL	BLM30$Urostomy problems	-0.34	<2.2e-16	Weak
C30$QL	Anchor_urological issues	-0.359	<2.2e-16	Weak
C30$QL	Anchor_sexual issues	-0.289	<2.2e-16	Weak
C30$QL	SHQ$sexual satisfaction	-0.24	<2.2e-16	Weak
C30$QL	SHQ$Sexual pain	-0.14	<2.2e-16	Very weak
C30$QL	C30$Nausea and vomiting	-0.226	<2.2e-16	Weak
C30$QL	C30$Appetite loss	-0.31	<2.2e-16	Weak
C30$QL	C30€$Constipation	-0.232	<2.2e-16	Weak
C30$QL	C30$Diarrhoea	-0.234	<2.2e-16	Weak
C30$QL	Anchor_gastrointestinal issues	-0.331	<2.2e-16	Weak
C30$QL	Fatigue	-0.62	<2.2e-16	Strong
C30$QL	SL (IN) insomnia	-0.285	<2.2e-16	Weak
C30$QL	PRO_55_A (Discouraged, F)	-0.48	<2.2e-16	Moderate
C30$QL	PRO_55_B (Discouraged, S)	-0.49	<2.2e-16	Moderate
C30$QL	PRO_55_C (Discouraged, I)	-0.51	<2.2e-16	Moderate
C30$QL	PRO_56_A (Sad, F)	-0.495	<2.2e-16	Moderate
C30$QL	PRO_56_B (Sad, S)	-0.501	<2.2e-16	Moderate
C30$QL	PRO_56_C (Sad, I)	-0.464	<2.2e-16	Moderate
C30$QL	PRO_46_A (Concentration, F)	-0.467	<2.2e-16	Moderate
C30$QL	PRO_46_B (Concentration, I)	-0.499	<2.2e-16	Moderate
C30$QL	PRO_47_A (Memory, F)	-0.334	<2.2e-16	Weak
C30$QL	PRO_47_B (Memory, I)	-0.378	<2.2e-16	Weak
C30$QL	PRO_54_A (Anxiety, F)	-0.351	<2.2e-16	Weak
C30$QL	PRO_54_B (Anxiety, S)	-0.344	<2.2e-16	Weak
C30$QL	PRO_54_C (Anxiety, I)	-0.323	<2.2e-16	Weak
